# Mind-Wandering during Personal Music Listening in Everyday Life: Music-Evoked Emotions Predict Thought Valence

**DOI:** 10.3390/ijerph182312321

**Published:** 2021-11-24

**Authors:** Liila Taruffi

**Affiliations:** Music Department, Durham University, Durham DH1 3RL, UK; liila.taruffi@durham.ac.uk

**Keywords:** mind-wandering, music-evoked emotions, visual mental imagery, mood regulation, health, wellbeing, digital health interventions, experience sampling method, personal music listening

## Abstract

Research has shown that mind-wandering, negative mood, and poor wellbeing are closely related, stressing the importance of exploring contexts or tools that can stimulate positive thoughts and images. While music represents a promising option, work on this topic is still scarce with only a few studies published, mainly featuring laboratory or online music listening tasks. Here, I used the experience sampling method for the first time to capture mind-wandering during personal music listening in everyday life, aiming to test for the capacity of music to facilitate beneficial styles of mind-wandering and to explore its experiential characteristics. Twenty-six participants used a smart-phone application that collected reports of thought, mood, and emotion during music listening or other daily-life activities over 10 days. The application was linked to a music playlist, specifically assembled to induce positive and relaxing emotions. Results showed that mind-wandering evoked during music and non-music contexts had overall similar characteristics, although some minor differences were also observed. Most importantly, music-evoked emotions predicted thought valence, thereby indicating music as an effective tool to regulate thoughts via emotion. These findings have important applications for music listening in daily life as well as for the use of music in health interventions.

## 1. Introduction

Music’s capability to influence ongoing conscious thought has not been systematically explored yet. Although some mental phenomena such as autobiographical memory [1], involuntary musical imagery (or “earworms” [2]), and music-evoked narratives [3] have received some attention from music researchers, very little is known regarding other types of internally-oriented cognition, such as mind-wandering. The purpose of this study was to explore the prevalence and main characteristics of mind-wandering evoked during personal music listening in daily life and, in particular, its relationship with the emotions evoked by music (personal music listening was defined here as listening to music alone, e.g., at home, or in a public space using a mobile device and headphones).

Mind-wandering is an omnipresent mental phenomenon consisting of a shift of attention away from the external world or an ongoing activity to an internally-oriented dynamic flow of thoughts and/or images. Such thoughts can vary in their degree of intentionality (with mind-wandering being deliberate or spontaneous), meta-awareness (being aware of our mind-wandering), task-relatedness (the degree to which mind-wandering can be associated with the task at hand, if there is any), their modality (the form in which mind-wandering occurs, for instance, images, thoughts, or sounds), and, of course, phenomenological content (for example, one can think about the future, self-relevant matters, other people, etc.) [4,5,6]. Experience sampling studies in both laboratory and daily life have provided a plethora of insights regarding mind-wandering’s frequency, e.g., [7], the contexts in which it unfolds [8], its neural underpinnings [9], and its effects on a wide range of psychological processes, [10]. For example, we know that humans can mind wander up to 50% of their waking time (which has also been translated into ~2000 mind-wandering episodes in a typical 16-h day; [11]), and that mind-wandering varies throughout the day (with lower rates early in the day, peaking at midday, and declining gradually before rising once more in the evening; [12]). While mind-wandering leads to incubation effects on creativity [13] and confers great mental freedom, allowing the planning of personally relevant future goals [14], it also carries significant costs to task performance [15,16]. For instance, mind-wandering during complex tasks that demand continuous attention, such as reading, is frequent and linked to detrimental effects on comprehension [17]. By contrast, the beneficial effects of mind-wandering are more likely to arise during tasks that require fewer cognitive resources [18]. However, monotonous and slow auditory signals [19] as well as boring or unpleasant tasks [20] are also known to increase mind-wandering.

A positive relationship between mind-wandering, negative mood, and poor wellbeing or mental health has been well documented by the literature. Individuals with depressive symptomatology (both clinically diagnosed and/or with tendencies to depression or neuroticism) exhibit increased levels of mind-wandering at both state and trait levels [21,22,23,24,25,26]. Similarly, negative mood triggers more frequent mind-wandering featuring personal concerns, negatively-hued thoughts, and past events [27,28]. In a seminal experience sampling study based on a sample of 2250 participants, Killingsworth and Gilbert [8] found that mind-wandering is an antecedent of negative mood and concluded that a wandering mind inevitably leads to unhappiness. A series of studies have followed from this initial finding to further examine the complex relationship between mind-wandering and mood states and highlighted that the impact of mind-wandering on subsequent mood highly depends on the content of people’s thoughts. For example, Ruby et al. [29] discovered that the emotional content of mind-wandering is linked to a corresponding valence of subsequent mood and that this direct relationship is modulated by the socio-temporal content of the thoughts (e.g., thoughts that are other- and past-related are linked to subsequent negative mood, even if current thought content is positive). Similarly, Poerio, Totterdell, and Miles [30] found that mind-wandering predicts negative mood only when thought content is *sad* or *anxious*, but not when *happy* or *calm*; other aspects of the content, such as a thought being *interesting*, are linked to positive subsequent mood [31]. Furthermore, other experiential characteristics of mind-wandering, such as intentionality and meta-awareness, might also play an important role in mediating its effects on wellbeing and mental health. Some preliminary evidence shows that people who more frequently engage in spontaneous mind-wandering are more likely to report symptoms of depression, anxiety, and stress, and that deliberate mind-wandering may buffer against these types of affective dysfunctions [32]. Likewise, high meta-awareness levels seem to ameliorate the detrimental effects of mind-wandering to ADHD symptomatology [33], and trait brooding (a thinking style typical of rumination that encapsulates the passive and judgmental pondering of one’s mood [34]) positively correlates with mind-wandering without meta-awareness [35]. In summary, these findings suggest that the relationship between mind-wandering, mood, and wellbeing is rather complex and is modulated by many factors, including the content of the thoughts (e.g., the thoughts being positive or negative), the personal characteristics of the mind-wanderer (e.g., a predisposition to depression), possibly some experiential dimensions of mind-wandering (e.g., intentionality and meta-awareness), and the nature of the task at hand (e.g., the task being boring or repetitive).

For many individuals, personal music listening is a common leisure activity that can provide a pleasurable and relaxed space for the mind to wander without leading to any detrimental effect on task performance. This is because the listener does not “perform” any task besides automatically processing the incoming auditory signal (obviously, this may not be true when music listening is a secondary or background activity, for example, when listening to music while driving or studying, or when dealing with focused/attentive music listening). In these “task-free” contexts, the emotional tone of the music could be harnessed to manipulate the content of listeners’ thoughts in order to favour a beneficial style of mind-wandering (i.e., one that features positive thought content and leads to subsequent positive mood), which could in the long-term boost overall wellbeing. Despite this unexplored potential of music to steer ongoing thoughts and images toward positive directions, research on music and mind-wandering is still scarce, with only a few studies published on this topic [36,37,38]. Nevertheless, this is clearly a crucial avenue for future mind-wandering research, given the ubiquity of this mental phenomenon and its strong impact on wellbeing and mental health [39] as well as the well-known power of music for mood regulation [40,41].

The first study on music and mind-wandering [36] made use of a thought sampling technique (i.e., intermittently probing individuals regarding their current mental state) in an online music listening task. We showed that music conveying and evoking sadness (in contrast to happiness) is associated with (*i*) an enhanced occurrence of mind-wandering, (*ii*) a corresponding content of thought (in both music conditions), and (*iii*) the engagement of the brain’s default mode network, which is typically involved in spontaneous cognition [42], among other networks [9,43]. Moreover, we also highlighted the predominance of visual mental imagery (compared with inner speech) as a modality through which mind-wandering episodes during music occur, regardless of the type of emotion experienced (sadness or happiness). In a following laboratory study employing heroic- and sad-sounding music [38], similar effects of the music on thought content were observed, but not on mind-wandering’s frequency. In particular, heroic-sounding music was linked to more positive, exciting, constructive, and motivating thoughts, while sad-sounding music was linked to calmer or more demotivating thoughts. A recent study provides stronger evidence of a close relationship between the music’s features and the contents of thought [44]. In this study, participants listened to different pieces of music while performing a directed imagination task that required watching a visual inducer in which a figure travels towards a barely visible landmark in the far distance, and then closing their eyes and imagine a continuation of the journey. After the imagined journeys, participants reported vividness, the imagined time passed and distance travelled, as well as the emotional tone of the imagined content. The results showed that vividness and emotional tone, as well imagined time passed and distances travelled, were systematically influenced by the music pieces. An earlier study [37] focused on daydreaming, a state of consciousness closely related to mind-wandering, which seems to involve a stronger visual imagery component [45,46] (note that visual mental imagery can be regarded both as a modality through which mind-wandering episodes occur and as an independent mental phenomenon). The authors found that daydreams arising in the course of the musical experience mediated the effect of the music’s type (sad vs. happy) on relaxation and liking. Specifically, sad music correlated with increased relaxation, whereas happy music promoted more positive daydreams, which in turn facilitated relaxation and were associated with participants’ increased liking of music. Interestingly, the findings from Martarelli et al. [37] underline that daydreams play a functional role when listening to music. Overall, the studies reviewed above highlight the potential of music to influence mind-wandering (and/or daydreaming) episodes, in particular with a capacity to modulate the content of these mental experiences and with a potential beneficial outcome in terms of mood, thought valence, and relaxation. However, these findings have limited ecological validity because the data were collected in the laboratory or during online tasks. As psychological (non-music) research on mind-wandering has shown, data gathered via experience sampling in daily life are important to identify the main characteristics of mind-wandering [8,47], and they exhibit similarities but also differences with laboratory data [48].

The aim of this study was to investigate the capability of music to evoke beneficial styles of mind-wandering in the context of everyday personal music listening, with a special focus on mind-wandering’s frequency, its main modality (visual mental imagery), its main experiential dimensions (valence, intentionality, and meta-awareness), and its relationship to mood (both antecedent and subsequent to the mind-wandering), as well as to music-evoked emotions. To capture mind-wandering episodes outside the lab, I employed the experience sampling method implemented via a smart-phone application (see 2. Materials & Methods). The application was linked to a music playlist (specifically prepared for this study to induce relaxing and positive affective experiences; for more detail see Section 2.5 Music stimuli) hosted by a music streaming provider (Spotify). It collected thought, emotion, and mood reports over a 10-day period, while participants listened to the music playlist or carried out other activities. For this study, participants were required to have two 5-min music listening episodes per day (see Section 2.3 Instructions for more detail regarding the listening task). During the ten days, the application also administered a series of psychometric questionnaires. Specifically, I sought to test the following hypotheses: First, I expected participants to mind-wander frequently (i.e., >50–55%) during the music listening in accordance with the averaged reported occurrence of mind-wandering of previous laboratory and online studies [36,37,38]. Second, I predicted that the selected playlist would be effective in enhancing participants’ moods [40]. Specifically, mood improvement was operationalised by an increase of the mean ratings of felt valence after the music listening. Participants were also expected to report moderate arousal ratings (i.e., around the midpoint of the scale) as the music stimuli were relaxing and calm. Third, because previous work suggests that music-evoked thoughts and emotions are entangled during the music listening [36,38], I expected that music-evoked emotions would predict thought valence. Fourth, I expected to corroborate (and extend to the music domain) previous findings showing that participants’ initial mood can predict thought valence, and inversely that thought valence and meta-awareness can impact the mood after the mind-wandering episode [29,30]. Finally, to explore whether music has a unique capability to shape mind-wandering episodes, data on participants’ thoughts (frequency, intentionality, valence, meta-awareness, and visual mental imagery) were compared between music and non-music episodes. There was no specific hypothesis in this regard, given the lack of information from previous research.

## 2. Materials & Methods

### 2.1. Participants

Twenty-six participants (18 female, *M_age_* = 30.46, *SD* = 9.63, 76.92% residing in UK) took part in the study and were recruited via Durham University student and staff mailing lists. Of the participants, 13 were students, 7 in full-time employment, 4 currently out of work, and 2 self-employed. The majority of participants reported to be music-loving non-musicians (15); among the rest, 6 were amateur musicians, 3 semi-professional musicians, and 2 professional musicians. Participants were required to: (*i*) have an android smart-phone, (*ii*) to carry it all the time for the length of the study, (*iii*) to have the Spotify application already installed in their smart-phones, and (*iv*) to regularly use the Spotify application for personal music listening. Participants received one GBP 10 Amazon voucher and also had the possibility to enter a prize draw to win one GBP 25 Amazon voucher.

### 2.2. Experience Sampling Protocol

The experience sampling methodology (ESM) [49,50] was used to obtain data on mind-wandering, mood, and music-evoked emotions. An updated version of MuPsych—a music-specific application for experience sampling with android smart-phones [51,52]—was employed in this study. This version of MuPsych was customised to suit the design of the current study.

MuPsych collected data via music experience sample reports (M-ESRs), non-music experience sample reports (NM-ESRs), and surveys. Participants were instructed to listen to the Spotify study playlist twice a day for 5 min each time. M-ESRs were presented immediately when the participant accessed their Spotify application (with a limit of one report per hour). If music was still playing on their phone after a period of 5 min—as automatically determined by MuPsych—a follow-up report would be presented. NM-ESRs were randomly notified four times daily and also led to a follow-up report that was notified by the application after 5 min. The duration of each report (both M-ESRs and NM-ESRs, and their follow-up reports) was about 1 min, and the duration of the completion of each survey (four in total) ranged approximately from 1 to 5 min. Participants were asked to regulate their awake times (e.g., 9.00 am–9.00 pm), so that the application would notify them only during the chosen hours.

### 2.3. Instructions

A few of days before the start of the study, participants received a set of instructions via email, which explained how they could download and install MuPsych on their phones and access the Spotify study playlist. Assistance via email was provided by the main researcher and the application creator to allow the correct functioning of the application.

Instructions on the music listening were provided at this stage and were also repeated in the home page of MuPsych. Specifically, participants were required to have two music listening episodes (of a minimum length of 5 min each) per day at a time of convenience. Each music episode could feature more than a music piece, depending on the total duration of each individual piece (see Section 2.5 Music stimuli). Participants were instructed to be alone, to relax as much as possible, to listen to the music without any interruption and on shuffle mode, and not to engage with other tasks that required focus (e.g., writing, working, etc.). It was clarified that during the course of the study participants were allowed to listen to the study playlist only twice a day. Nevertheless, they could listen to their own music on Spotify (data regarding these music episodes were also collected, but their analysis will be included elsewhere).

With regard to the completion of the experience sample reports (both M-ESRs and NM-ESRs), participants were instructed to take a mental snapshot of their mental and emotional experience at the moment just before receiving the notification from the application. The importance of filling out the report immediately after receiving the notification was also stressed. If responding to a notification immediately was not possible (for example, because the participant was driving or attending an important meeting), participants were asked to remember their answers and fill them out later. However, when answering the questions related to mind-wandering and music-evoked emotions (featured in the follow-up reports of the M-ESRs; see Section 2.4 Measures), participants were specifically instructed to concentrate on their mental and affective experiences during the entire length of the music listening (and not on the moment just before receiving the notification).

### 2.4. Measures

All items were presented in English. In the M-ESRs, the initial item assessed current mood, with responses given on two 7-point sliders, with the first being *valence* (with −3 = “negative”, 0 = “neutral”, and +3 = “positive”), and the second *arousal* (with −3 = “very low”, 0 = “moderate”, and +3 = “very high”). Participants additionally selected one *mood*, out of a list, that best matched their current affective state and rated how intensely they felt that mood (*intensity*) on a 7-point slider (with −3 = “not at all” and +3 = “very much so”). The following three items used a list-response format to assess where the participants were (*location*), what they were doing before starting the music listening (*activity*), and who they were with (*people*).

If music was still playing after 5 min, a follow-up report would be notified by MuPsych. The items featured in the follow-up reports assessed some of the continuous variables measured at the start of the music listening (*valence*, *arousal*, and *intensity*) to determine how they changed over the 5-min music listening episode. The rest of the items assessed mind-wandering, its main characteristics, music-evoked emotions, and familiarity with the music (familiarity was included to control for memories and implicit associations with the music). The presence or absence of *mind-wandering*, including visual mental imagery, was measured by response to the following question “What were you just thinking about?” (with answer options being “music”, “visual images (realistic or fantastical)”, or “something else”). Mind-wandering was considered *present* if participants selected “something else” or “visual images”, while it was considered *absent* when “music” was selected. Depending on the answer given to this first item, a number of questions inquiring into the nature of the participant’s thoughts followed, yielding a more fine-grained picture on thoughts and images. If “music” was chosen, participants were asked to further select one of the following options: “I was completely focused on the music”, “I was thinking about some aspects of the music (instrumentation, etc.)”, “I was evaluating the music (whether I like it or not)”, “other: please briefly specify” (with a randomised order of answer options). If “visual images” was chosen, the following item was presented “Were those images associated with the music?” (with answer options being “yes” or “no”). If “something else” was chosen, participants were asked to further select one of the following options: “I was thinking about everyday stuff”, “I was thinking about personal plans/goals”, “I was worrying about something”, “I was thinking about something from the past”, “other: please briefly specify” (with a randomised order of answer options). The *valence* of thoughts and visual images was measured by response to the following question “Was the content of your thoughts/images positive or negative?” (with −3 = “negative”, 0 = “neutral”, and +3 = “positive”). To capture the *intentionality* of mind-wandering, participants were asked to select one of the following options: ‘‘I allowed my thoughts to wander on purpose”, ‘‘I found my thoughts wandering spontaneously”, “I don’t know” (with a randomised order of answer options). *Meta-awareness* was measured with the following item “How aware were you of where your attention was focused?” (with −3 = “completely unaware” and +3 = “completely aware”). All the mind-wandering items described above were prepared based on previous research, see [30,36,53,54]. The final items assessed the *emotions* experienced during the music listening by employing the Geneva Emotional Music Scale (GEMS; [55]), and the *familiarity* with the music. The GEMS comprises nine categories (wonder, transcendence, tenderness, nostalgia, peacefulness, power, joy, tension, and sadness), which condense into the three main factors of *sublimity*, *vitality*, and *unease*. Participants were asked to rate how intensely they felt each of the nine emotions on a 5-point slider (with −2 = “not at all”, 0 = “moderately”, and +2 = “very much”). Following the GEMS ratings, participants were asked to indicate their familiarity with each randomly played music piece on a 5-point slider (with −2 = “I have never heard this piece before” and +2 = “I know this piece”).

The NM-ESRs and their follow-up reports featured the same items of the M-ESRs and their corresponding follow-up reports (with the exclusion of the GEMS and familiarity). Note that the item assessing the presence or absence of *mind-wandering* was adapted to fit a non-music context (see Table 2 for the text of this item).

Surveys included an assessment of wellbeing (Flourishing Scale (FS) [56]) and tendencies to affective disorders (Depression Anxiety Stress Scale (DASS) [57]). Participants also filled the Ten-Item Personality Inventory [58] and the Interpersonal Reactivity Index [59], but the data related to these questionnaires were not used in the present study and will be reported elsewhere. The FS is a brief 8-item measure of wellbeing, which describes important aspects of human functioning ranging from positive relationships to feelings of competence and having meaning and purpose in life. All items are phrased as positive statements, and the possible range of scores is from 8 to 56. High scores indicate that respondents view themselves positively regarding important areas of functioning or that they have many psychological resources and strengths. The DASS assesses the negative emotional states of depression, anxiety, and stress and yields a total of 21 items. The *depression* subscale assesses dysphoria, hopelessness, devaluation of life, self-deprecation, lack of interest/involvement, anhedonia, and inertia. The *anxiety* subscale assesses autonomic arousal, skeletal muscle effects, situational anxiety, and subjective experience of anxious affect. The *stress* subscale is sensitive to levels of chronic non-specific arousal. It assesses difficulty relaxing, nervous arousal, being easily upset/agitated, irritable/over-reactive, and impatient. Descriptive statistics for each of the used instruments (along with normative scores) are displayed in Table 1. The average score for the FS was 45.17 (*SD* = 7.09), indicating a very good level of wellbeing, which is in line with the normative data from student populations (*M* = 44.97, *SD* = 6.56 [56]). Regarding the DASS, the means for each subscale were within the recommended cut-off scores for conventional severity labels [57], thus the sample did not exhibit a general tendency to affective disorders.

### 2.5. Music Stimuli

The music stimuli featured in the study playlist were 41 instrumental pieces from ambient, electronic, post rock, classical, and film music, varying in length between 55 s and 10 min and 3 s (*M* = 3.93, *SD* = 2.31). Stimuli were selected based on their capability to induce positive and relaxing emotions as well as mind-wandering and visual mental imagery. Instrumental music was chosen over music with lyrics in order to keep the design as simple as possible because lyrics have been found to have effects on listeners’ emotion and imagery [60,61], which would have been difficult to tease apart from the music *per se*. It is nevertheless important for future work on this topic to extend the stimulus set to encompass music with lyrics. Some of these pieces were taken from my previous study on music and mind-wandering [36], while the rest were selected in a pilot study. In the pilot experiment, 45-s excerpts of 68 instrumental music pieces were rated by a sample of 146 volunteers (83 female). Each participant rated 17 excerpts out of the 68, presented in a randomised order. Participants were asked to rate their felt emotions on two 7-point scales of valence and arousal ranging from −3 (indicating a negative valence and a low arousal) to +3 (indicating a positive valence and a high arousal). To assess the capability of the selected pieces to stimulate mind-wandering and visual mental imagery, I used the same items presented in the ESM study (“What were you just thinking about?”, with answer options being “music”, “visual images (realistic or fantastical)”, or “something else”). The final set of 41 stimuli was associated with positive valence (*M* = 1.58, *SD* = 1.18) and moderate arousal (*M* = 0.41, *SD* = 1.22). Moreover, mind-wandering occurred in 51% of the music, with 27% being thoughts unrelated to the music (“something else”) and 24% visual images.

The familiarity ratings gathered in the subsequent ESM study indicated that participants were overall unfamiliar with the selected music pieces (*M* = −0.64, *SD* = 1.68). The full list of music pieces is available in the Supplementary Materials (Table S1).

### 2.6. Data Analyses

Because the data had a natural two-level structure, in which responses collected over a series of time-points (report-level units) were nested within individuals (person-level units), a generalized linear mixed-effects model approach was adopted to examine the effects of: (*a*) music-evoked emotions on the valence of thoughts; (*b*) initial mood on the valence of thoughts; and (*c*) thought valence and meta-awareness on final mood. Linear mixed-effects models are an extension of linear regression and have the advantage of being able to retain all measurements from each participant (without aggregating them), leading to more statistical power and less unexplained variance [62,63], and they have been successfully applied to similar ESM research on mind-wandering [53]. This analysis was implemented in STATA.

Given the exploratory nature of this research question, for the comparison of the experiential characteristics of mind-wandering between music and non-music episodes, an aggregate approach was chosen. Aggregate scores were created for each participant, producing descriptive and inferential stats for the main variables of interest. This approach has been utilized in previous ESM studies using the MuPsych application [52,64].

## 3. Results

### 3.1. ESR Response Rates

Overall, M-ESRs were completed on 433 occasions and their follow-up reports on 226 occasions, corresponding to a 52.19% response-rate (i.e., the percentage of started M-ESRs that were then completed). NM-ESRs were completed on 442 occasions and their follow-up reports on 303 occasions, corresponding to a higher response-rate of 68.55%. The difference in response-rates between M-ESRs and NM-ESRs is probably due to the fact that the music M-ESRs required more time commitment and attention from the participants compared with NM-ESRs, mostly due to the music listening “task”.

### 3.2. Location, Activity, and People

The context of each recorded report (regardless of whether music was present or not) was operationalised by assessing the physical location, the main activity that the participants were engaged with before they were notified by the application, and the presence of other people. Only descriptive statistics are reported for these variables because they were not related to any hypothesis or aim of the study (a full inferential analysis of these data is beyond the scope of this article). To give a general overview of the locations, activities, and people where the ESRs occurred, the relative frequency of these variables across music and non-music episodes is reported below and illustrated in the Supplementary Materials (Figures S1–S3).

The most frequent locations during the music episodes were home (57.37%), travelling (20.74%), and work (13.13%) (Figure S1). The same locations were also most frequent in the non-music episodes (home = 54.52%, travelling = 9.73%, work = 16.29%), although travelling was not so common as during music listening. For both music and non-music episodes, all the remaining locations were reported below 5%, with the exception of school/university for NM-ESRs (8.14%).

Overall, the most frequent main activities were working/studying (19.71%), walking (9.98%), relaxing/meditating (9.74%), doing nothing/waiting (8.79%), being on the bus/train/plane (8.31%), grooming/self-care (6.89%), and focused music listening (5.70%) (Figure S2). All the remaining activities were reported below 5%. The most frequent main activities during the non-music episodes were working/studying (25.92%), eating (10.55%), doing nothing/waiting (7.11%), gaming/entertainment (6.19%), web-browsing (5.96%), relaxing/meditating (5.50%), and walking (5.50%). All the remaining activities were reported below 5%.

The final feature of the context concerned other people being present during the ESR (Figure S3). The overall most frequent condition was alone in both music (only me/headphones = 53.69%, only me/speakers = 42.40%) and non-music episodes (49.32%). However, in non-music episodes, participants also reported being accompanied by colleagues/workmates (14.93%), partner/spouse (12.44%), friends (10.86%), family members (7.47%), and strangers (4.98%). Percentages for these conditions in the music episodes were all between 0–1% due to the nature of the listening instructions given to participants (see Section 2.3 Instructions in the Materials & Methods).

### 3.3. Rate and Phenomenology of Mind-Wandering

Table 2 summarises the observed rates of mind-wandering, visual mental imagery, and music-related thoughts. Mind-wandering occurred in 57% of all sampled music episodes. This total frequency breaks down into 38% being thoughts unrelated to the music (“something else”) and 19% visual images (realistic or fantastical). In the large majority of the sampled music episodes, participants reported that their visual imagery was associated with the music (63%) rather than not associated (37%). Thoughts regarding the music amounted to 43%. Furthermore, mind-wandering occurred in 36% of all the randomly sampled, non-music episodes.

Table 3 presents the characteristics of mind-wandering during M-ESRs and NM-ESRs as reported by the participants. Thoughts regarding everyday stuff were frequent in both music (42%) and non-music (39%) episodes and were followed by worries (music = 19%; non-music = 12%), personal plans (music = 18%; non-music = 39%), and memories from the past (music = 10%; non-music = 9%). These thought profiles did not significantly differ between M-ESRs and NM-ESRs (*p* > 0.05), with the only exception of the thought category of personal planning, which was significantly more pronounced during non-music episodes (χ^2^(3) = 8.97, *p* = 0.03).

### 3.4. Main Experiential Dimensions of Mind-Wandering: Thought Valence, Intentionality, and Meta-Awareness

Overall, thoughts had a positive valence during both M-ESRs (*M* = 0.71, *SD* = 1.48; Table 3) and N-ESRs (*M* = 0.56, *SD* = 1.67; Table 3), and both mean ratings were significantly higher than the neutral mid-point of the valence scale (music: *t* (98) = 4.75, *p* < 0.001; non-music: *t* (105) = 2.88, *p* < 0.005). Thought valence tended to be slightly more positive during music episodes, but this difference was not significant (*p* > 0.05).

Regarding the intentionality of mind-wandering episodes, spontaneous thoughts were more frequent in both music (65%) and non-music episodes (59%) compared with deliberate thoughts (music = 31%; non-music = 29%) and not being able to answer this question (music = 4%; non-music = 12%) (Table 3). Furthermore, there was no significant association between the intentionality of thought (spontaneous, deliberate) and the type of ESRs (music, non-music), as shown by a chi-square test (χ^2^(1) = 0.76, *p* > 0.05).

Participants reported to be relatively aware of their thoughts during both M-ESRs (*M* = 0.52, *SD* = 1.29; Table 3) and N-ESRs (*M* = 0.92, *SD* = 1.54; Table 3), and meta-awareness was significantly higher during non-music episodes (*t* (424) = 3.05, *p* < 0.005). For both music and non-music episodes, meta-awareness was significantly higher during deliberate mind-wandering (music: *M* = 1.29, *SD* = 1.32, *t* (93) = 4.09, *p* < 0.001; non-music: *M* = 1.64, *SD* = 1.24, *t* (140) = 5.37, *p* < 0.001) than spontaneous mind-wandering (music: *M* = 0.02, *SD* = 1.35; non-music: *M* = 0.28, *SD* = 1.49).

Similarly, thought valence was more positive during deliberate mind-wandering (music: *M* = 0.84, *SD* = 1.37; non-music: *M* = 1.02, *SD* = 1.62) than spontaneous mind-wandering (music: *M* = 0.64, *SD* = 1.50; non-music: *M* = 0.42, *SD* = 1.57); however, this difference was slightly significant only for non-music episodes (*t* (140) = 2.10, *p* < 0.037).

### 3.5. Mood Change

**Figure 1:**
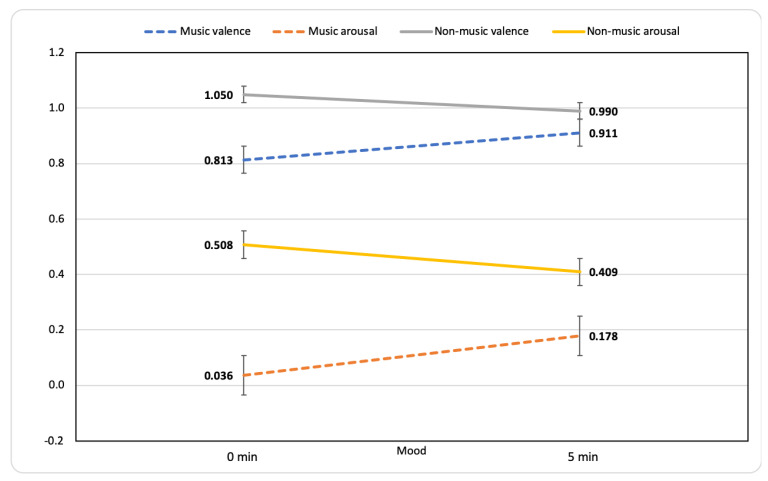
Mood changes across music and non-music episodes. Mean ratings (±SEM) for valence and arousal are illustrated at min 0 (first report) and min 5 (follow-up report). Dotted lines indicate a significant difference between ratings at min 0 and min 5. Valence and arousal answer scales ranged from −3 (indicating a negative valence and a low arousal) to +3 (indicating a positive valence and a high arousal), see Section 2.4 Measures.

Music listening was effective at improving participants’ moods, as indicated by the significant difference between the ratings of valence and arousal assessed before (valence: *M* = 0.81, *SD* = 1.35; arousal: *M* = 0.03, *SD* = 1.53) and after (valence: *M* = 0.91, *SD* = 1.16, *t* (224) = −1.705, *p* = 0.04; arousal: *M* = 0.18, *SD* = 1.44, *t* (224) = −2.67, *p* = 0.004; Figure 1) the 5-min music episodes. These results show an increasing trend for valence and arousal ratings, reflecting a moderate level of arousal (i.e., around the midpoint of the scale) and a clearer positive valence.

Regarding N-ESRs, valence and arousal ratings actually decreased over the 5-min gap between the first and the follow-up report. However, such changes were not significant (valence: *t* (302) = 1.17, *p* = 0.24; arousal: *t* (302) = 1.64, *p* = 0.06; Figure 1).

**Figure 2:**
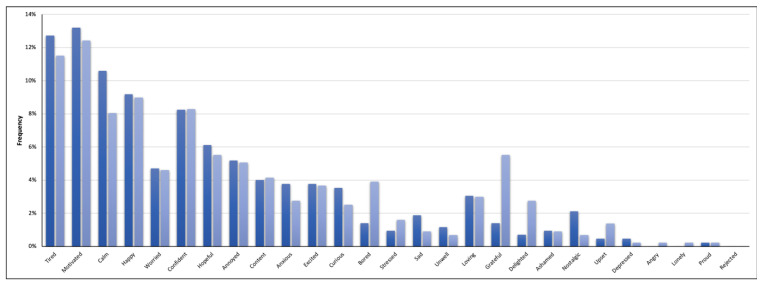
Frequency of initial moods in music episodes (dark blue bars) and non-music episodes (light blue bars).

Figure 2 shows the relative frequency (in percent) of the reported initial moods during music and non-music episodes. These results suggest fairly similar trends, regardless of the condition. For instance, during both M-ESRs and NM-ESRs the moods “tired”, “motivated”, “calm”, “happy”, and “confident” were the most common, and the moods “depressed”, “lonely”, “angry”, “proud”, and “rejected” were the least common. Nevertheless, some differences are also noticeable. Specifically, the moods “bored”, “grateful”, and “delighted” were significantly more frequent during the non-music (*vs.* music) episodes (bored: χ^2^(1) = 5.26, *p* = 0.02; grateful: χ^2^(1) = 10.80, *p* = 0.001; delighted: χ^2^(1) = 5.40, *p* = 0.02).

Means for the intensity of the initial and final moods during music episodes are reported in Table 4. Interestingly, there was a reduction in the intensity ratings for the moods “tired”, “bored”, “annoyed”, “worried”, “calm”, and “curious” after the 5-min music listening episode. Inversely, there was an increase in the intensity ratings for the moods “confident”, “content”, and “hopeful”. However, from all the reported changes, only those ones related to the moods “bored”, “annoyed”, “worried”, and “calm” were significant (bored: *M_1_* = 0.80, *SD_1_* = 0.45, *M_2_* = −0.60, *SD_2_* = 1.14, *t* (4) = 2.33, *p* = 0.039; annoyed: *M_1_* = 1.15, *SD_1_* = 0.69, *M_2_* = 0.23, *SD_2_* = 0.72, *t* (12) = 3.49, *p* = 0.002; worried: *M_1_* = 1.00, *SD_1_* = 0.39, *M_2_* = 0.36, *SD_2_* = 0.84, *t* (13) = 2.86, *p* = 0.007; calm: *M_1_* = 1.31, *SD_1_* = 0.67, *M_2_* = 0.63, *SD_2_* = 1.34, *t* (18) = 2.23, *p* = 0.038; Table 4). A decreasing trend of tiredness levels among participants was observed, although it did not reach significance (*M_1_* = 1.45, *SD_1_* = 0.76, *M_2_* = 1.22, *SD_2_* = 0.82, *t* (36) = 1.60, *p* = 0.059; Table 4).

### 3.6. Prevalence of Specific Music-Evoked Emotions

Table 5 shows the mean intensity ratings of the nine GEMS emotions experienced during the music listening episodes. A repeated-measures ANOVA was conducted on these ratings for each emotion to identify the most intense emotional experience for the listeners. A significant main effect of the type of emotion was found (*F* (7, 686) = 18.81, *p* < 0.0001). Bonferroni pairwise comparisons confirmed that peacefulness (*M* = 0.57, *SD* = 1.21) was the most intensely felt emotion, exhibiting significant differences with all the remaining emotions, excluding nostalgia (*M* = 0.18, *SD* = 1.79; *p* = 0.16). Inversely, tension (*M* = −1.24, *SD* = 1.33) was the least intensely felt emotion as indicated by Bonferroni pairwise comparisons, which showed significant differences with all the remaining emotions (including sadness; *M* = −0.74, *SD* = 1.46; *p* = 0.002). These results reflect the emotional tone of the selected music and the targeted music listening experience.

Interestingly, thought valence ratings exhibited significant positive correlations (moderate to large effect sizes; see Table 5) with ratings of positively valenced felt emotions, such as power (*r* = 0.32, *p* < 0.01), joy (*r* = 0.64, *p* ≤ 0.001), tenderness (*r* = 0.37, *p* ≤ 0.001), and peacefulness (*r* = 0.46, *p* ≤ 0.001).

Similarly, thought valence ratings exhibited significant negative correlations (moderate to large effect sizes; see Table 5) with ratings of negatively valenced felt emotions such as tension (*r* = −0.5, *p* ≤ 0.001) and sadness (*r* = −0.4, *p* ≤ 0.001).

### 3.7. Do Music-Evoked Emotions Predict Thought Valence?

A linear mixed-effects model with the nine music-evoked emotions (wonder, transcendence, tenderness, nostalgia, peacefulness, power, joy, tension, and sadness) as predictors and thought valence as the dependent variable showed a significant overall effect, *x*^2^ [9, 99] = 149.21, *p* < 0.0001. Specifically, the emotions that made a significant contribution to the model were sadness, ß = −0.16, SE = 0.08, z = −1.95, *p* = 0.04, 95% CI [−0.33, 0.001]; tenderness, ß = 0.27, SE = 0.11, z = 2.41, *p* = 0.016, 95% CI [0.05, 0.49]; tension, ß = −0.21, SE = 0.09, z = −2.39, *p* = 0.017, 95% CI [−0.38, −0.04]; joy, ß = 0.42, SE = 0.13, z = 3.31, *p* = 0.001, 95% CI [0.17, 0.68]; and power, ß = 0.18, SE = 0.08, z = 2.29, *p* = 0.022, 95% CI [0.026, 0.34].

### 3.8. Does Initial Mood Predict Thought Valence?

A linear mixed-effects model with the initial valence and initial arousal as predictors and thought valence as the dependent variable showed a significant overall effect, *x*^2^ [2, 99] = 43.56, *p* < 0.0001. Further analysis found that only initial valence made a significant contribution to the model, ß = 0.54, SE = 0.13, z = 4.05, *p* < 0.001, 95% CI [0.28, 0.79].

### 3.9. Do Thought Characteristics Predict Final Mood?

A linear mixed-effects model with thought characteristics (valence and meta-awareness) as predictors and final valence as the dependent variable showed a significant overall effect, *x*^2^ [2, 99] = 38.90, *p* < 0.0001. Further analysis found that only thought valence made a significant contribution to the model, ß = 0.50, SE = 0.08, z = 5.90, *p* < 0.001, 95% CI [0.33, 0.67].

Another linear mixed-effects model with thought characteristics (valence and meta-awareness) as predictors and final arousal as the dependent variable did not show any significant effect, *x*^2^ [2, 99] = 4.81, *p* = 0.09.

## 4. Discussion

One of the crucial functions of music, which is documented cross-culturally, is its power to evoke intense emotions and to regulate moods. What so far has received less attention by researchers is the relationship between internally-oriented thoughts (and images) and affective experiences that are elicited during music listening. This study employed the experience sampling methodology, implemented via a music-based smartphone application, to explore mind-wandering episodes during personal music listening in everyday life. The application collected real-time and ecologically valid reports of thought, mood, and emotion while participants listened to a wide range of music pieces capable of evoking positive and relaxing emotions; the application also collected non-music reports randomly throughout the day. This approach allowed testing for the capability of music to favour beneficial styles of mind-wandering, which were defined here as featuring positive thought content and leading to subsequent positive mood. In addition, the study aimed to provide an exploratory investigation of the experiential characteristics of mind-wandering during music in daily life because the available data, from a yet small literature body, are based mostly on online or laboratory experiments, employing a limited range of music genres (typically classical and film music). This study instead made use of a broader range of music genres, including instrumental pieces from ambient, electronic, post rock, classical, and film music, which were mostly unfamiliar to the participants.

### 4.1. Rate and Phenomenology of Mind-Wandering

With respect to the rate of mind-wandering during music episodes, this study found that mind-wandering (including thoughts unrelated to the music and visual mental images) occurred in 57% of the sampled reports. This result (*i*) is in line with my first hypothesis; (*ii*) corroborates earlier findings by Taruffi et al. ([36]; 53% during sad music and 47% during happy music), Cooper, Acomb, and Ma ([65]; 50% during sad music and 46% during happy music; this is a small-scale, student replication study of Taruffi et al. [36]), and Deil et al. ([66]; 64% during experimental and ambient live music); and (*iii*) extends such rates to daily life settings. However, Martarelli et al. [37] reported lower mind-wandering rates (38.8% during instrumental music), while Koelsch et al. [38] reported higher ones (72.6% during heroic music and 73.7% during sad music), suggesting fluctuations that may be due to differences in music styles, individual characteristics of the listeners, contextual variables, and conceptualisation of mind-wandering and related assessment methods (on this last point, please see Section 4.6 Limitations and directions for future research). Notably, when adopting a “strict” definition of mind-wandering (including only thoughts unrelated to the music and excluding visual mental imagery), this new estimate amounts to 38%, which is very similar to those of previous non-music ESM studies measuring mind-wandering evoked in various daily life contexts and reporting mind-wandering at almost one third of the signals, e.g., [20,30,67,68]. Moreover, this last estimate is also in line with the frequency rate of mind-wandering during non-music episodes (36%) as observed in this study.

Further inspection of the phenomenology of participants’ mind-wandering episodes reveals similar thought profiles during both music and non-music episodes. For example, thoughts regarding everyday matter were very common among the participants in both M-ESRs and NM-ESRs. Memories and worries were also reported, but less frequently. Although autobiographical memories are commonly cued by familiar and autobiographically salient music [69], this study made used of researcher-selected music stimuli, which were unfamiliar to the participants. This provides an explanation for the low rates of memories evoked during music listening (although familiarity and preference with these music genres were not assessed and should be considered in the future). Despite the overall correspondence of thought categories during M-ESRs and NM-ESRs, thoughts regarding personal plans and goals were more often reported during non-music episodes than music episodes, pointing to a potential unique route in which music may impact mind-wandering. It is in fact possible that music, which always unfolds in the present moment, may hold the listener to a present-centred perspective, thereby reducing those forms of thinking that are future-related, such as thinking about personal plans and goals. This is obviously highly speculative but is nevertheless supported by the common use of music in mindfulness practises and represents an issue to be further explored by future studies.

Visual mental imagery was also experienced by the participants in line with previous work that has stressed its relevance during music listening [70,71]. What strikes most in this study is that almost the double of the occurrences of visual mental images were somewhat associated with the music rather than being independent from it, underscoring the capability of music to guide corresponding visual imagery [3,44,72].

### 4.2. Main Experiential Dimensions: Thought Valence, Intentionality, and Meta-Awareness

Thought valence was positive during both music and non-music episodes, and this trend was slightly more pronounced (although the difference was not significant) during music listening. Positively-hued thoughts and images during music were also found by most of the previous studies [36,37,38,44], despite the fact that the emotional tone of some of the selected music pieces was negative (however, in Koelsch et al. [38], sad music induced slightly negative thought valence). This may suggest that music, regardless of its emotional tone, can often exert a positive influence on the thoughts and images simply because it is very often an enjoyable activity *per se*.

In considering the intentionality of mind-wandering, participants reported the large majority of their thoughts to be spontaneous rather than deliberate for both music and non-music episodes (with no significant difference between them). This result seems to go against a general trend found in daily life (non-music) studies, which reveals a prevalence of deliberate vs. spontaneous mind-wandering [73]. However, our result is in line with a recent (music) study investigating the characteristics of mind-wandering during a live music event [66], and therefore may reflect a specific feature of mind-wandering occurring in music contexts more broadly. Further studies will need to investigate how intentionality levels vary within listening settings, motivations for listening, enjoyment of the music, and complexity of the music, among other factors.

Notably, participants exhibited moderate levels of awareness of where their attention was during both music and non-music episodes, and meta-awareness was significantly higher during NM-ESRs. These results have interesting implications for research on wellbeing because meta-awareness is well known for being positively related to mindfulness [74]. Nevertheless, the possibility that meta-awareness was induced by the type of assessment employed in this study should at least be considered. Asking participants to reflect on their own inner experiences may indeed enhance or require participants to be aware of their thoughts during the experiment. Further studies could try to clarify this point by employing self-caught methods, where participants have to report each time they notice their minds wander rather than reporting when probed, as in the probe-caught method employed in this study.

Deliberate (vs. spontaneous) mind-wandering was associated with stronger meta-awareness (in both M-ESRs and NM-ESRs) and more positive thought valence (only in NM-ESRs). Such findings are in line with the assumption that meta-awareness is needed to deliberately direct mind-wandering, while it is not necessary when thoughts wander spontaneously [75]. Moreover, given that deliberate mind-wandering requires conscious attentional effort, it makes sense that it is more often linked to positive thoughts.

### 4.3. Mood Regulation and Emotion Evocation via Music

The selection of instrumental music used in this study was capable of enhancing participants’ mood in a 5-min timeframe, as shown by the significant increase in valence ratings in line with my second hypothesis (although this effect was small). Arousal ratings also significantly increased (probably due to the music’s activation), but they still reflected moderate levels of energy in line with the relaxing tone of the music. Such enhancement of valence and arousal did not occur during the non-music episodes, thereby stressing the effectiveness of music for mood regulation, even when the music is not self-selected by the participants. Furthermore, during music listening episodes, a number of negative moods, such as, e.g., “annoyed” and “worried”, exhibited a significant decrease in their intensity, suggesting a successful regulation of negative moods. The role of music for mood regulation, which typically encompasses the improvement of negative moods and emotions, the reductions of distress, and the enhancement of positive moods and emotions, is supported by a wide range of previous literature, including survey studies, e.g., [40,41,76,77], as well as ESM or diary studies in daily life [64,78,79]. Interestingly, people in Western countries report that mood regulation is one of the primary motivations for engaging with music in daily life [40,80], and music listening was found to be the second most successful activity (with the first being exercise) aimed at repairing a negative mood as well as boosting arousal and releasing tension [81].

When looking at the specific emotions evoked during music listening, participants reported mostly moderately arousing, pleasurable, and aesthetic affective experiences such as peacefulness, nostalgia, tenderness, and wonder (with peacefulness being the most frequently evoked emotion). This was in line with the study’s goal of inducing a positive and relaxing affective experience in the participants that could in turn create a comfortable personal space for the mind to wander in positive directions.

### 4.4. Thought Valence Regulation via Music-Evoked Emotions

The main focus of this research was to examine, in more detail compared to previous work, the relationship between mind-wandering and music-evoked emotions. It is noteworthy that music-evoked emotions predicted thought valence in line with my third hypothesis. Specifically, the emotions of sadness and tension predicted negatively valenced thought, while tenderness, joy, and power predicted positively valenced thought (with joy accounting for 4.2% of the variance). The correlation analysis between the intensity ratings of the GEMS emotions and the ratings of thought valence also corroborated these findings and the role of each individual emotion highlighted above (with the exception of peacefulness, which despite exhibiting a strong positive correlation was not a significant predictor of thought valence). The observed correspondence between the valence of both thought and emotion is in line with previous studies on music and mind-wandering, which, mainly by means of qualitative analysis, found a relationship between the tone of the music and the one of the reported thoughts [36,38]. The present study, by employing linear mixed-effects model analyses and experience sampling in daily life, provides a much stronger and ecologically valid evidence as well as a broader generalizability of the findings to everyday life personal music listening settings and to a wider variety of music genres. Importantly, these findings demonstrate that music-evoked emotions can systematically influence thought valence, thereby pointing to emotion as a possible mechanism underlying “thought regulation”.

Interestingly, the “sublime” subset of the GEMS emotions (i.e., wonder, nostalgia, transcendence, and peacefulness, with the exception of tenderness) did not significantly predict thought valence ratings, whereas the “vitality” (i.e., power and joy) and “unease” (i.e., sadness and tension) subsets were successful predictors. One could thus suggest that the so-called “sublime” emotions induce a nuanced valence that difficultly translates into a clearly positive or negative thought valence. On the other hand, “vitality” and “unease” feature emotions with a much clearer valence (either positive or negative) that may influence thought valence in a more straightforward fashion. The fact that a marked positive or negative emotional valence has a stronger potential to influence thought valence constitutes an important indication for music listening habits in daily life and future music-based interventions. More specifically, the present findings suggest that listening to joyful music would help to shift thoughts towards positive directions more effectively than music evoking aesthetic emotions.

### 4.5. Thought’s Relationship with Prior and Subsequent Mood

One final focus of the study was to replicate previous findings on the relationship between mind-wandering and mood both prior and subsequent to the mind-wandering episode. The present data indeed revealed that initial mood (in particular, the reported valence) predicted thought valence and vice versa that thought valence predicted subsequent mood (again, only valence). This is in line with my fourth hypothesis and a number of studies demonstrating a close connection between mood and mind-wandering’s phenomenological characteristics, including valence [29,30]. In contract to my hypothesis, meta-awareness did not predict subsequent mood. However, this result does not rule out the possibility that meta-awareness may play a role in healthy styles of cognition, as has emerged in previous literature [74], but it may simply indicate that it is not relevant for short-term thought regulation.

### 4.6. Limitations and Directions for Future Research

The present research features several limitations that should be acknowledged. First, the lack of a unified theoretical framework that can clearly distinguish between related, inner experiences with music (e.g., visual mental images, daydreaming, thoughts unrelated to the music, memories, future-related thoughts, etc.) has clearly hindered the operationalisation of mind-wandering in the present study as well as in the entire previous literature on this topic. In this study, I adopted a “family-resemblances” approach to mind-wandering (as proposed by Seli et al. [73]), which considers mind-wandering as a multidimensional construct held together by patterns of overlapping and nonoverlapping features (e.g., meta-awareness, intentionality, etc.), thereby accounting for the heterogeneity of mind-wandering experiences. The construct of mind-wandering was operationalised here by employing multiple items that could separate between the different types of thought and its dimensions. However, it remains necessary for music psychologists to engage with further theoretical efforts to encourage a more nuanced and rigorous understanding of the different types of internally-oriented mental experiences with music in a similar way to what has been done among psychologists and philosophers [73,82,83]. Adapting existing theories on mind-wandering or mental imagery to music contexts may be an effective way to push this area of research further (for an example of a music-specific framework to understand visual mental images, see [70]).

Although my results are the first to provide an insight into the phenomenology of mind-wandering as it occurs during personal music listening in daily life, such findings are still preliminary because they are limited by the small sample size. Moreover, the data may reflect the individual thinking styles of the participants, for example, a propensity to experience specific thought patterns such as thinking about everyday matter. A large-scale correlational study examining the links between personality characteristics and mind-wandering content patterns should be able to shed light on this point. Despite the current study initially aimed for a much larger sample size, the data collection was interrupted by the outbreak of the COVID-19 pandemic. Furthermore, a cross-cultural approach would be valuable to shed light on the impact of culture in shaping thoughts, images and emotions during music listening [3,84], as the current study only included Western participants. To advance knowledge on this topic, it would also be important to analyse “app-data”. To a certain extent, the data reported in this study can be considered as “task-data”, because participants had to follow specific instructions when listening to the Spotify music playlist. A focus on “app-data” would enhance the in vivo aspect of the study (i.e., exploring mind-wandering as naturally occurring in everyday life) and therefore its overall ecological validity.

Another limitation of this study concerns the characterisation of mind-wandering episodes. This study opted to focus on a few selected dimensions, such as valence, intentionality, and meta-awareness, to keep the ESRs very brief given that the study was already quite long (10 days). However, other thought characteristics that were not assessed here (e.g., temporal focus) may have played a role, especially in modulating mood after the mind-wandering episode, as shown by previous research [29]. In addition, it would be important for future work to establish the phenomenology of participants’ thoughts also in a bottom-up fashion and not exclusively by imposing a structure based on previous findings (as in this study). In this sense, working with open-ended participants’ reports and data-driven analysis techniques, such as principal component analysis or thematic analysis, may hold a stronger potential to unveil the underlying low-level phenomenological structure of ongoing thoughts [85]. Identified themes could then be further explored in connection with personality data to test the relative extent to which music and personality can shape them.

This study found that music-evoked emotions predict the valence of thought; however, the present results yield no information regarding the temporal and causal relationships between the evoked emotions and thoughts. In fact, one difficult question to tackle by future research regards the dynamic interaction between the affective and cognitive processes during music listening. Recent work has suggested that emotional responses to music occur before visual mental imagery [86]; this would fit well with the present results, but it would also contradict previous theories of music-evoked emotions [87]. Future work exploring the causal and temporal relationships between cognitive and affective phenomena will be pivotal to clarify this issue and may consider that the order of the various phenomena could depend on the characteristics of the task and the conscious attention of the listener, as well as the nature of the induced affective experiences. For instance, Vroegh [88] observed a unidirectional effect from emotion to imagery when explicitly positive emotions are evoked, while mixed emotions were linked to a reversed effect, from imagery to felt emotions.

The current study provides preliminary data and materials to design a protocol for a music-based digital intervention aimed at regulating thoughts via music. Smartphone interventions have been increasingly trialled in recent years and hold many advantages, including that they can be easily tailored to the participant’s needs and provide a more comfortable private environment that can be accessed anonymously, at a time and location of choice [89,90]. Mindfulness applications are very common among the domain of mental health and wellbeing, and meta-analyses have started to explore the effectiveness of mindfulness-based interventions [91]. With regard to music, there are only a few studies attesting the effectiveness of music for the regulation of mood and distress via smartphone applications [64,92]; however, none of them has explored the regulation of thoughts and/or mental states.

Finally, a pivotal area of expansion of this research involves music-based interventions in clinical populations. The present study featured a healthy sample of participants, as supported by the scores on the FS and DASS; therefore, it is currently unknown whether the current findings would extend to a clinical sample. In general, music research has shown differences in music enjoyment, selection, and mood regulation strategies between healthy and depressed individuals [93,94,95]. The capability of music to modulate thought could be harnessed by evidence-based therapies (see, for example, imagery rescripting [96]), which successfully employ imagination as a clinical tool [44,70]. In this sense, my findings call for a systematic examination of the effectiveness of music-based therapies for mental health conditions that feature dysfunctional styles of cognition such as rumination in depression or intrusive imagery in post-traumatic stress disorder.

## 5. Conclusions

In a unique effort to examine music’s capability to modulate thought in everyday life, this study found that music-evoked emotions (in particular, joy) predict thought valence of mind-wandering episodes during personal music listening. This means that the continuous build-up of affective states driven by the music has important implications for internally-oriented mental states such as mind-wandering, and stresses the interdependence between cognitive and affective processes, which are both inherent in music listening. Given that participants’ mind-wandering featured an overall positive thought valence, and that thought valence predicted the valence of mood after the music episode, these results imply that music can be effectively harnessed, beyond mood regulation, as a tool to reinforce positive styles of mind-wandering. Importantly, the observed pattern of results supports the view that emotion acts as a route through which music modulates ongoing thought. Overall, the findings provide fruitful insights and novel directions into music’s potential to maintain wellbeing for both listeners and therapists.

## Figures and Tables

**Table 1:** Participant background measures (including means and standard deviations) and normative data.

	Study Sample (*N* = 26)		Normative Data	
	*M*	*SD*	*M*	*SD*
*DASS*				
Depression	8.40	3.90	7.19	6.54
Anxiety	6.96	4.29	5.23	4.83
Stress	13.04	3.94	10.54	6.94
*FS*	45.17	7.09	44.97	6.56

*Note*. *DASS* = tendencies to affective disorders; *FS* = wellbeing. Normative data are from Lovibond and Lovibond (1995; *DASS*) and Diener et al. (2010; *FS*).

**Table 2:** Breakdown of the focus of attention during music and non-music episodes.

**Music Episodes:** “What were you just thinking about?”	
*Music*	43%
I was thinking about some aspects of the music (instrumentation, etc.)	64%
I was completely focused on the music	16%
I was evaluating the music (whether I like it or not)	20%
*Visual images (realistic or fantastical)*	19%
Images were associated with the music	63%
Images were not associated with the music	37%
*Something else*	38%
**Non-Music Episodes:** “Are you thinking about something other than what you’re currently doing?”	
*No*	64%
*Yes*	36%

**Table 3:** Experiential characteristics of mind-wandering during music and non-music episodes.

	Music Episodes	Non-Music Episodes
*Thoughts*		
“I was thinking about everyday stuff”	42%	39%
“I was worrying about something”	19%	12%
“I was thinking about personal plans/goals”	18%	39%
“I was thinking about something from the past”	10%	9%
Other	10%	1%
*Intentionality*		
“I allowed my thoughts to wander on purpose”	31%	29%
“I found my thoughts wandering spontaneously”	65%	59%
“I don’t know”	4%	12%
*Thought valence* (*M* ± *SD*)	0.71 (1.48)	0.56 (1.67)
*Meta-awareness* (*M* ± *SD*)	0.52 (1.29)	0.92 (1.54)

*Note*. “Other” includes thoughts about work and international and national news.

**Table 4:** Initial and final intensity ratings of individual moods during the music episodes.

Mood	Intensity		Change
	Min 0	Min 5	
curious	1.86	1.29	−0.57
content	1.75	1.83	+0.08
confident	1.62	1.92	+0.31
motivated	1.57	1.57	0
excited	1.57	1.57	0
happy	1.50	1.50	0
tired	1.45	1.22	−0.24
calm	1.31	0.63	−0.68 *
annoyed	1.15	0.23	−0.92 **
worried	1.00	0.36	−0.64 **
hopeful	1.00	1.08	+0.08
anxious	0.82	0.82	0
bored	0.80	− 0.60	−1.40 *

*Note*. Increasing (+) and decreasing (−) trends are indicated in the column labelled “change”. * *p* < 0.05, ** *p* < 0.01. Moods are ordered from the most to the least intensely experienced. Answer scales ranged from −3 (indicating a low mood intensity) to +3 (indicating a high mood intensity), see Section 2.4 Measures.

**Table 5:** Mean (*SD*) of the intensity ratings for each GEMS emotion evoked during the music (ordered from the least to the most experienced) and their correlations with the valence of thought.

Emotion	*M* (*SD*)	*r*
tension	−1.24 (1.33)	−0.5 ***
sadness	−0.74 (1.46)	−0.40 ***
power	−0.64 (1.29)	0.32 **
transcendence	−0.29 (1.50)	0.12
joy	−0.21 (1.35)	0.64 ***
wonder	−0.06 (1.45)	0.09
tenderness	−0.02 (1.50)	0.37 ***
nostalgia	0.18 (1.79)	0.13
peacefulness	0.57 (1.21)	0.46 ***

*Note.* ** *p* < 0.01, *** *p* ≤ 0.001. Ratings for felt emotions were given on a 5-point scale from −2 = “not at all” to +2 = “very much”, see Section 2.4 Measures.

## Data Availability

The data is available by request to the author.

## Supplementary Materials

The following are available online at https://www.mdpi.com/article/10.3390/ijerph182312321/s1, Table S1: Music pieces features in the Spotify playlist; Figure S1: Locations where the ESRs took place as a function of music and non-music episodes; Figure S2: Main activities as a function of music and non-music episodes; Figure S3: People as a function of music and non-music episodes.
