# Supplementary material for: Mind-Wandering during Personal Music Listening in Everyday Life: Music-Evoked Emotions Predict Thought Valence
Source: Int J Environ Res Public Health. 2021 Nov 24;18(23):12321. doi: 10.3390/ijerph182312321 (PMC8656507; doi:10.3390/ijerph182312321)
Supplement: Supplementary file 1 [file ijerph-18-12321-s001.zip › ijerph-1432308-supplementary.pdf]

Supplementary Materials

Table S1. Music pieces features in the Spotify playlist.

Title	Artist
A Walk	Tycho
Aether	Hildur Guõnadóttir
Ambre	Nils Frahm
Anakin's Theme	John Williams
Anandi	Mychael Danna
Appa's Lesson	Mychael Danna
Arrival of the Birds/Exodus	The Cinematic Orchestra
Cambridge, 1963	Jóhann Jóhannsson
Da Nessuna Parte	Paolo Cognetti
Daydream	Nitin Sawhney
Dawn	The Cinematic Orchestra
Divenire	Ludovico Einaudi
Divorce Papers	Arcade Fire and Owen Pallett
Dream 3 (in the midst of my life)	Max Richter
Dream 13 (minus even)	Max Richter
Gabriel's Oboe	Ennio Morricone
Griet's Theme	Alexandre Desplat
Hoppipolla	Sigur Rós
Impossible Island	Gaussian Curve
Infra 5	Max Richter
Julia	Ludovico Einaudi
Love Theme	Ennio Morricone
Lullaby from the Westcoast Sleepers	Max Richter
Luminous	Max Richter
Once Upon a Time in America (Deborah's Theme)	Ennio Morricone
Re	Nils Frahm
Says	Nils Frahm
The Black Dog and the Scottish Play	Hilmar Örn Hilmarsson
The Imitation Game	Alexandre Desplat
The John Dunbar Theme	John Barry
The Prize of One's Life	James Horner
The Theory of Everything	Jóhann Jóhannsson
Them	Nils Frahm
Truman Sleeps	Philip Glass, Burkhard Dallwitz
Turning Page (Instrumental)	Sleeping at Last
Un Altro Sguardo	Paolo Cognetti
Una Mattina	Ludovico Einaudi
Wayward Sisters	Abel Korzeniowski
Welcome Chris	Andrea Guerra
Wrong Floor	Cliff Martinez
Your Hand in Mine	Explosions in the Sky

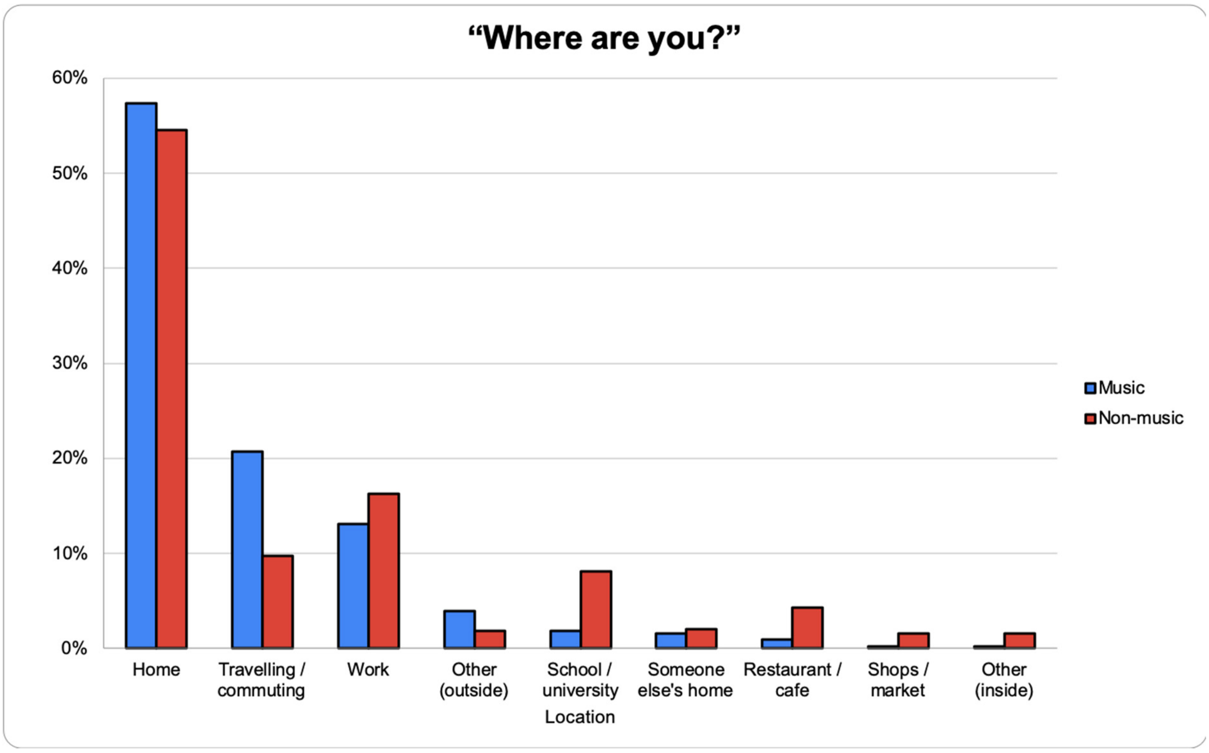

Figure S1. Locations where the ESRs took place as a function of music and non-music episodes.

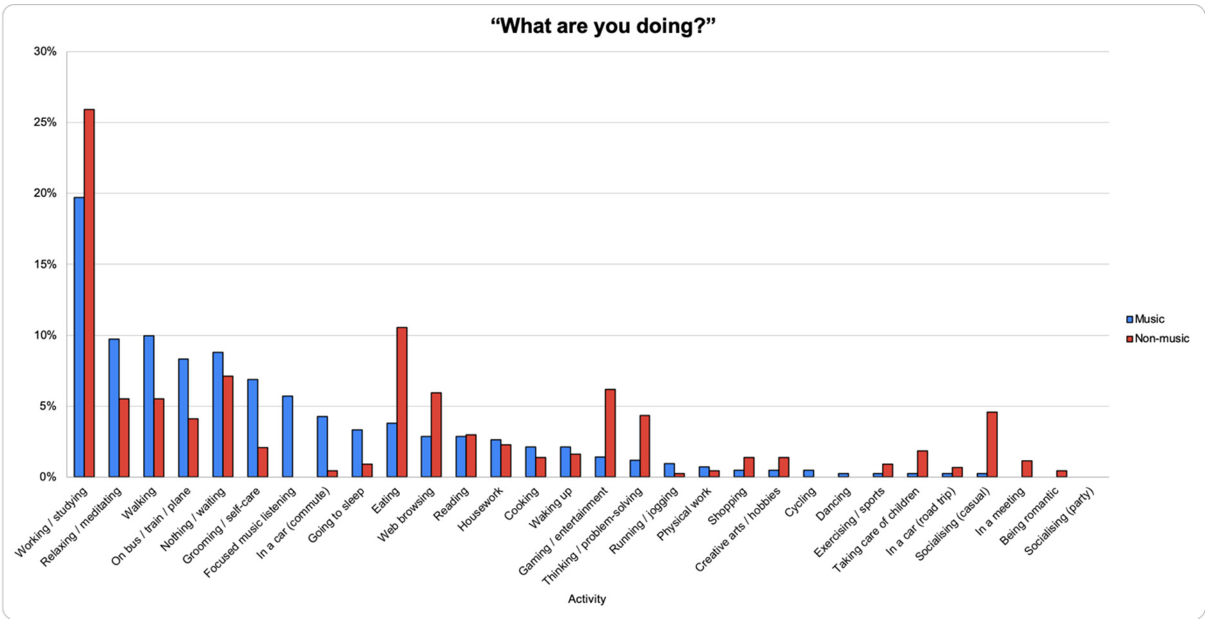

Figure S2. Main activities as a function of music and non-music episodes.

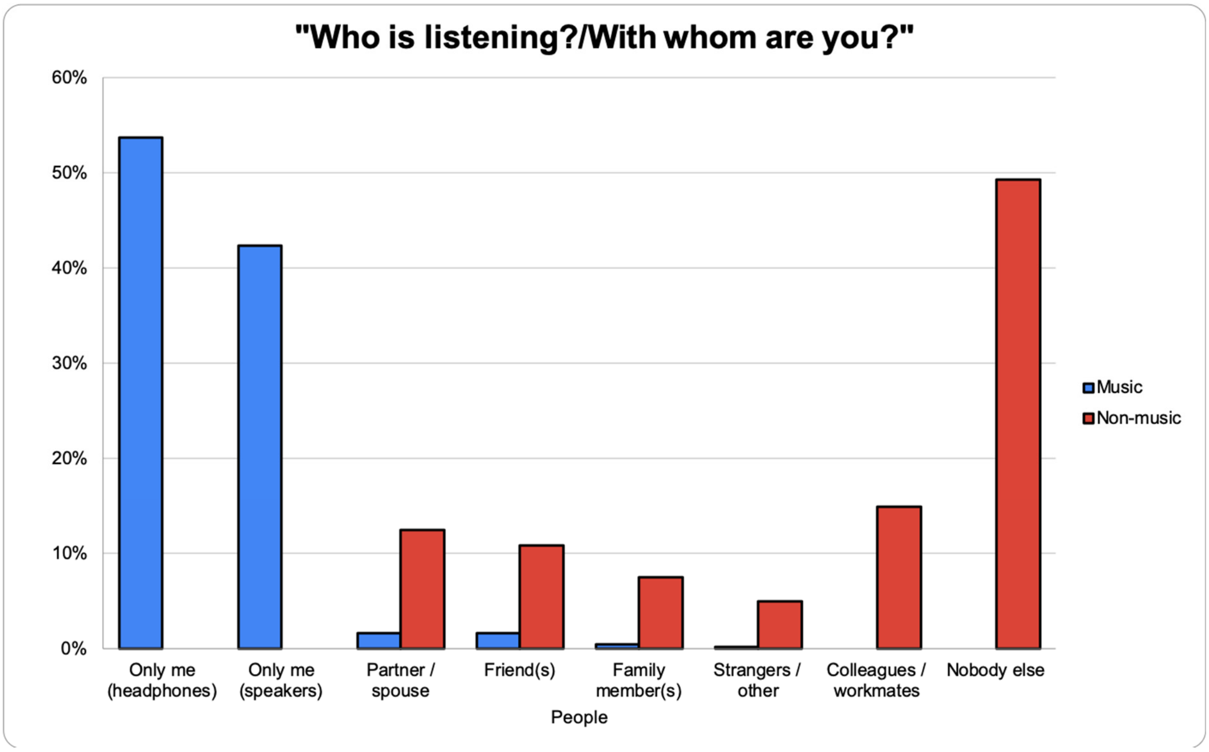

Figure S3. People as a function of music and non-music episodes.
